# First and second transscleral cyclophotocoagulation treatments provide similar intraocular pressure-lowering efficacy in patients with refractory glaucoma

**DOI:** 10.1007/s10792-022-02234-4

**Published:** 2022-02-03

**Authors:** Enrico Bernardi, Marc Töteberg-Harms

**Affiliations:** 1 Medical Faculty, University of Zurich, Zurich, Switzerland; 2 Department of Ophthalmology, University Hospital Zurich, Frauenklinikstrasse 24, 8091 Zurich, Switzerland; 3 Medical College of Georgia, Department of Ophthalmology, Augusta University, Augusta, GA USA

**Keywords:** Glaucoma, Cyclophotocoagulation, G-Probe, CPC, CW-TSCPC

## Abstract

**Purpose:**

The aim of this study is to address the safety and effectiveness of a second continuous-wave transscleral cyclophotocoagulation (CW-TSCPC) treatment by comparing its outcome against a first CW-TSCPC treatment in the same patients with refractory glaucoma.

**Methods:**

Twenty-one eyes with either primary or secondary glaucoma received a second CW-TSCPC laser session ≥ 3 months after the first treatment. Intraocular pressure (IOP), best-corrected visual acuity (BCVA), and number of topical or oral ophthalmic pressure-reducing medications were registered at every time point up to the last follow-up at 3 months. A complete slit-lamp examination was conducted to record for complications or other abnormal ocular findings. Success was defined as IOP between 6 and 21 mmHg and > 20% reduction in IOP with or without anti-glaucoma medications.

**Results:**

At 3 months follow-up of the first CW-TSCPC treatment, a 24.8% decrease in IOP was observed, whereas a 45.6% IOP decrease was observed 3 months post the second CW-TSCPC treatment. Visual acuity did not decrease, and no major complications were observed post either treatment within the follow-up period. Time to failure was 79.5 ± 24.6 and 77.1 ± 29.4, respectively (*P* = 0.955). No serious complications were observed.

**Conclusion:**

A second CW-TSCPC treatment proved to be a safe and effective treatment option when the first CW-TSCPC treatment was insufficient in maintaining the desired IOP level for a prolonged time (mean time between both sessions 6.4 ± 8.0 months).

## Introduction

Glaucoma is a chronic, progressive optic neuropathy with a multi-factorial etiology [1–5]. It is the leading cause of irreversible blindness worldwide [6–8]. An irreversible damage of the optic nerve and a progressive loss of nerve fibers causes vision loss [9–11]. However, an appropriate and timely therapy can effectively prevent progressive nerve damage, loss of visual field, and thus blindness [12–15]. In most cases, glaucoma is associated with a pathological increase of intraocular pressure (IOP) (> 21 mmHg), and, therefore, therapies are aimed at reducing IOP [16, 17].

There are several ways of achieving IOP reduction: (1) medical treatment (mostly topical eye drops), (2) surgical procedures (incisional surgeries and minimally invasive glaucoma surgery (MIGS) procedures), or (3) lasers (i.e., laser trabeculoplasty, endocyclophotocoagulation, cyclophotocoagulation) [18–21].

Continuous-wave transscleral cyclophotocoagulation (CW-TSCPC) using the G-Probe® delivery device (IRIDEX Corp., Mountain View, CA, USA) is an established procedure that achieves IOP reduction through destruction of the pigmented cells within the ciliary body to decrease aqueous humor production [22–26]. This approach uses energy administered with an 810 nm continuous-wave laser [27, 28]. The energy is absorbed by the melanin present in the pigmented epithelium of the ciliary body and leads to an increase in temperature, which causes coagulation. A “pop” sound can be heard once coagulation is reached in the target pigmented epithelium. The energy should be kept just below this “pop” level [29]. Unfortunately, this modality also increases thermal elevation to the surrounding tissue causing a high degree of collateral damage. In addition, it is known that there is variation in the location of the pigmented epithelium of the ciliary body from eye to eye, and within one eye, which causes unpredictability of treatment success to some degree. Common complications of CW-TSCPC are persistent intraocular inflammation, hyphema, cystoid macular edema, decreased visual acuity or vision loss, and persistent hypotony (phthisis bulbi) [30]; hence, its use is usually limited to refractory glaucomas.

Patients sometimes require a second CW-TSCPC treatment to achieve the desired IOP reduction and successfully retain it over time. In previous studies, the retreatment rate ranged from 0 to 81% [31–35]; however, numerous CW-TSCPC retreatments come with an increased risk of side effects. Previously treated eyes may tend to react to the coagulative laser energy with an excessive inflammatory response. Given the factors involved with reoperations, the rate at which they occur has been proposed as an indicator of the quality of care and a marker of surgical quality [36, 37]. Managing complications may have social, occupational, and financial consequences to the patient and society while also increasing the surgical cost and clinical burden for the hospital or clinic. This is especially true within the first 90 days, which is the time frame most insurance companies consider as the postoperative “global period,” or the time considered by where no additional reimbursement will be paid to manage complications [38].

The efficacy of subsequent CW-TSCPC retreatment has not been widely addressed in the literature and, thus, is the aim of this study.

## Materials and methods

This is a retrospective, comparative interventional chart-review of patients with various diagnosis of glaucoma who received two treatment sessions of CW-TSCPC at the University Hospital Zurich, Zurich, Switzerland. The cantonal ethics commission of Zurich granted its approval to the study protocol and the study follows the principles of the Declaration of Helsinki and international and national laws. As stated in the protocol and according to the Art. 34 Human Research Act, the patients considered for this study signed either a general consent for research before the operation or an adapted consent specific for this study.

### Inclusion and exclusion criteria

Surgeries were performed between 03/2016 and 01/2020. Patients selected for the study had either primary or secondary glaucoma and were diagnosed with moderate to advanced glaucoma. Patients had prior, failed glaucoma surgery and non-controlled IOP on maximum tolerated meds (refractory glaucoma) or had advanced glaucoma with poor BCVA (i.e., ≤ 0.2 Decimal Snellen) and/or advanced, central involving visual field defects and non-controlled IOP on maximum tolerated meds. Patients who were underage at the time of the operation or who did not provide their agreement with either the general consent or the study-specific consent were automatically excluded from the study. In addition, patients were excluded when the second CW-TSCPC treatment was performed less than 3 months after the first.

### Procedure, anesthesia, and postoperative care

All procedures were performed by one glaucoma specialist (MTH). Immediately prior to CW-TSCPC, all patients received intravenous analgesia (50 mg fentanyl) and sedation (1–1.5 mg/kg body weight thiopental sodium 0.5 g/20 ml) under monitored anesthesia care. The G-Probe handpiece was used with the IRIDEX Cyclo G6™ Laser System (Iridex Corp., Mountain View, CA, USA). The Cyclo G6 was set in its continuous-wave treatment mode, and power was set to 2000 mW for 2500 ms per laser spot. Fifteen laser spots were applied, sparing the superior aspect of the globe from 10 to 2 o’clock. Two percent methylcellulose (Methocel, OmniVision, Puchheim, Germany) was used to guarantee a liquid interface. Care was taken to avoid the 3 o’clock and 9 o’clock meridians, areas of scleral thinning, sites of filtering blebs, and sites of glaucoma drainage devices. As the same protocol was used for the first CW-TSCPC treatment, treatment locations would potentially be at the same location where a laser spot was applied during the first treatment. If a “scleral spot” was seen from the first laser treatment, the spot during the second treatment was placed slightly apart from the visible scleral spot. After the treatment, the eye was patched for 24 h with a fixed combination ointment of tobramycin 3 mg/ml plus dexamethasone 1 mg/ml (Tobradex ointment; Alcon, Fort Worth, TX, USA). The next day, patients were started on unpreserved dexamethasone 1 mg/ml eye drops 5x/d for 1 week (Dexafree UD eye drops; Théa PHARMA SA, Schaffhausen, Switzerland) and unpreserved ofloxacin eye drops 3x/d for 3 days (Floxal UD eye drops; Bausch & Lomb Swiss AG, Zug, Switzerland). All patients were instructed to continue with their preoperative anti-glaucoma medication regimen. Medical hypotensive treatment was adjusted for each patient at every visit and was reduced, whenever possible, in a stepwise approach and at the surgeon’s discretion.

### Baseline and follow-up data collection

To evaluate and compare the efficacy of the first versus the second CW-TSCPC treatments, data collected at baseline included age at the time of surgery, sex, type of glaucoma, number of glaucoma medications (topical and oral ophthalmic pressure-reducing medications; meds), IOP (obtained through Goldmann applanation tonometry), best-corrected visual acuity (BCVA), and spherical equivalent. The participants underwent follow-up visits at 1 day, 1 week, 1 month, and 3 months postoperatively. At each postoperative appointment, the following factors were registered: spherical equivalent, IOP, BCVA, and meds. Simultaneously, a complete slit-lamp examination was conducted to record the following complications: corneal edema, persistent ocular hypotony (i.e., IOP ≤ 5 mmHg) on two consecutive follow-up visits, choroidal detachment, phthisis bulbi, sympathetic ophthalmia, cystoid macular edema, or any other abnormal ocular findings. A loss of vision of two or more lines in BCVA compared to baseline or a loss of light perception vision was also noted and considered as a complication.

### Statistical analyses

Excel 2016 was used for data management and IBM SPSS Statistics (International Business Machines Corporation (IBM), Armonk, NY, USA) version 26 was used for statistical analysis. Descriptive statistics were reported as mean ± SD for continuous variables and as absolute values and percentage for categorical variables. Preoperative and postoperative data were compared using student’s t test for equality of means (continuous variables) and chi-square test (categorical variables). A *P* value of < 0.05 was considered to be statistically significant. Differences in survival were assessed by Kaplan Meier survival statistics. Differences in survival between the two groups were computed using Log Rank (Mantel-Cox) statistics. Success was defined as either an IOP between 6 and 21 mmHg at the last visit and an IOP reduction of > 20% compared to the baseline measurement.

## Results

In total, 21 eyes of 21 patients were treated with CW-TSCPC two consecutive times within the time frame of the study. The mean time between both CW-TSCPC sessions was 6.4 ± 8.0 months. The average age was 64.7 ± 16.5 years; gender was 71.4% males and 28.6% females; eyes were 33.3% right and 66.7% left; and the diagnosis was 14.3% primary open-angle glaucoma, 42.9% pseudoexfoliative glaucoma, and 42.9% other glaucoma types (Table 1). The mean baseline IOP was 35.1 ± 16.3 mmHg before the first CW-TSCPC treatment and 31.4 ± 12 mmHg before the second CW-TSCPC treatment.

**Table 1:** Demographical data

	CW-TSCPC 1 and 2
Eyes	42 OD 14 (33.3%) OS 28 (66.7%)
Mean age [years]	64.8 ± 16.2 years
Gender	30 males (71.4%) 12 females (28.6%)
Spherical Equivalent	-2.82 ± 3.5
Baseline BCVA	2.7 ± 0.3
Baseline IOP [mmHg]	33.2 ± 7.3
Baseline AGD	2.8 ± 1.1
*Diagnosis* Primary open-angle glaucoma Pseudoexfoliative glaucoma Other glaucomas	6 (14.3%) 18 (42.9%) 18 (42.9%)

*OD* right eye, *OS* left eye, *BCVA* best-corrected visual acuity, *logMAR* logarithm of the minimum angle of resolution, *IOP* intraocular pressure, *mmHg* millimeters of mercury, *VF MD* visual field mean defect, *dB* decibel

The first CW-TSCPC treatment achieved a 34.3% reduction in IOP compared to baseline at day one (*P* < 0.001), 43.4% at 1 week (*P* < 0.001), 29.8% at 1 month (*P* < 0.001), and 24.8% at 3 months (*P* = 0.009). The second CW-TSCPC treatment achieved an IOP reduction of 13.2% 1 day postoperatively (*P* = 0.308), 48.3% at 1 week (*P* = 0.317), 32.2% at 1 month (*P* = 0.459), and 45.6% at 3 months (*P* = 0.023). Notably, no loss of vision and no other serious complications occurred after either treatment. At baseline, meds were slightly higher at the time of the first CW-TSCPC treatment than at the time of the second treatment (3.0 ± 1.4 vs. 2.5 ± 1.5; *P* = 0.249). The decrease in meds at 3 months was 21.3% after the first treatment and 14.4% following the second treatment (*P* = 0.991). At 3 months, there was a significant difference in IOP reduction between the two groups, with the second CW-TSCPC treatment showing a greater decrease in IOP (45.6 vs. 24.8%, *P* = 0.001). The baseline IOP was slightly lower among patients before the second CW-TSCPC treatment (31.4 ± 12 mmHg) compared to the first CW-TSCPC treatment (35.1 ± 16.3 mmHg) (Table 2). Time to failure after the first CW-TSCPC was 79.5 ± 24.6 days and 77.1 ± 29.4 after the second CW-TSCPC treatment. No statistically significant difference in success between the first CW-TSCPC and the second CW-TSCPC treatments was found (*P* = 0.955).

**Table 2:** Preoperative and postoperative data for BCVA, IOP, and Meds

	BCVA [logMAR]			IOP [mmHg]			Medications		
	1st CW-TSPC	2nd CW-TSPC	*P*	1st CW-TSPC	2nd CW-TSPC	*P*	1st CW-TSPC	2nd CW-TSPC	*P*
Baseline	2.8 ± 3.8	2.7 ± 3.6	0.975	35.1 ± 16.3	31.4 ± 12.0	0.403	3.1 ± 1.4	2.5 ± 1.5	0.249
1 day	2.8 ± 3.7	4.3 ± 4.4	0.319	23.1 ± 11.3	27.2 ± 13.4	0.308	0	0	-
1 week	3.4 ± 4.2	4.4 ± 4.5	0.472	19.9 ± 13.7	16.2 ± 8.5	0.324	2.7 ± 1.4	2.6 ± 1.6	0.781
1 month	2.7 ± 3.7	3.4 ± 4.2	0.595	24.6 ± 15.0	21.3 ± 13.0	0.457	2.5 ± 1.6	2.4 ± 1.5	0.783
3 months	1.0 ± 1.0	3.3 ± 4.1	< 0.001	26.4 ± 13.0	16.7 ± 6.8	0.001	2.4 ± 1.4	2.1 ± 1.4	0.991

*BCVA* best-corrected visual acuity, *logMAR* logarithm of the minimum angle of resolution, *IOP* intraocular pressure, *mmHg* millimeters of mercury

## Discussion

Based on the findings of this study, a second CW-TSCPC proved to be effective in terms of survival of success and more effective in terms of lowering IOP compared to the first CW-TSCPC treatment. Both procedures achieved a significant decrease in IOP compared to preoperative IOP at all visits. At the same time, meds were significantly reduced and were lower at all visits in comparison to baseline, with the exception of 1 week after the second procedure. Kaplan–Meier survival did not differ significantly between the first and second CW-TSCPC treatments; therefore, a second CW-TSCPC treatment is non-inferior to a first treatment. No serious complications occurred during the follow-up period of this study.

Potentially, a second CW-TSCPC treatment could be less effective compared to the first treatment. The hypothesis is that during the second CW-TSCPC treatment, the laser would be applied at the same locations of the ciliary body as the primary treatment, which have already been coagulated. Thus, no additional decrease in aqueous humor production by the second laser spots can be expected since the tissue in this location has already been destroyed. Despite this consideration, the presented data demonstrate comparable effectiveness between first and second CW-TSCPC treatments.

This finding is crucial since the first CW-TSCPC treatment sometimes does not lower IOP sufficiently to reach a preset target pressure or suffice in maintaining a desirable IOP level for a prolonged time. A second laser treatment being as effective as a first treatment grants ophthalmologists more options when deciding how to further lower IOP when the first operation proved to be not effective enough; otherwise, more invasive treatments, e.g., tube shunts, must be discussed with the patients. In our data, we did not find a higher risk for hypotony after two CW-TSCPC interventions. However, sample size and follow-up time are limited and, thus, this complication may have been missed. The cumulative energy reached after the second operation is ≤ 150 Joules (2 CW-TSCPC treatment sessions each with 15 laser spots, each laser spot was performed at 2′000 mW and for 2′500 ms), which could still be considered safe enough and not enhance the risk of hypotony. Aujla et al. found the cumulative energy after CW-TSCPC as a risk factor for hypotony. They found a cutoff of > 190 Joules for eyes developing hypotony, while eyes which received less cumulative energy did not develop hypotony [39].

## Conclusion

The second CW-TSCPC treatment for patients with refractory glaucoma demonstrated safe and effective and non-inferior to the first CW-TSCPC treatment. Therefore, a second CW-TSCPC treatment should be considered a valid option when trying to further lower IOP after a first CW-TSCPC treatment failed to achieve the target IOP.

## Data Availability

The data supporting this study’s findings are available from the corresponding author upon reasonable request and within national and international laws.
