# Correction to: First and second transscleral cyclophotocoagulation treatments provide similar intraocular pressure-lowering efficacy in patients with refractory glaucoma

**DOI:** 10.1007/s10792-022-02249-x

**Published:** 2022-04-25

**Authors:** Enrico Bernardi, Marc Töteberg-Harms

**Affiliations:** 1 Medical Faculty, University of Zurich, Zurich, Switzerland; 2 Department of Ophthalmology, University Hospital Zurich, Frauenklinikstrasse 24, 8091 Zurich, Switzerland; 3 Department of Ophthalmology, Medical College of Georgia, Augusta University, Augusta, GA USA

## Correction to: Int Ophthalmol https://doi.org/10.1007/s10792-022-02234-4

In the original publication, the given and family names of authors were published incorrectly. The correct version is updated in this correction.

The original article has been corrected.

